# Implementation of the Norwegian school meal guideline: Development and reliability of two questionnaires to measure adherence

**DOI:** 10.1177/1403494820972590

**Published:** 2020-12-10

**Authors:** Jorunn S. Randby, Helene Holbæk, Nanna Lien

**Affiliations:** 1 Department of Nutrition, Institute of Basic Medical Sciences, University of Oslo, Norway; 2 Department of Child and Adolescent Health, Norwegian Directorate of Health, Norway

**Keywords:** Adherence, after-school services, guideline, Norway, monitoring, questionnaires, reliability, school food, schools

## Abstract

*Aims:* This article reports on the development and reliability of two questionnaires that measure adherence to the Norwegian National Guideline on Food and Meals in School among primary schools and after-school services. *Methods:* Questionnaires for school principals and after-school leaders were developed systematically, using the following steps: (a) selection of scope, questions and adherence values; (b) face validity testing through expert review of initial drafts; (c) content validity testing through 19 cognitive interviews; (d) assessment of test–retest reliability in samples of principals (*n* = 54) and after-school leaders (*n* = 47); and (e) development of adherence indices. *Results:* The cognitive interviews led to substantial revisions of the draft questionnaires, increasing content validity through improved relevance and clarity. Test–retest assessment showed that Cohen’s κ ranged from −0.03 to 1.0 for principals and from −0.05 to 0.98 for after-school leaders, with 64 and 53% of values rated as ‘substantial’ or better. Percentage agreement averaged 85% among principals and 82% among after-school leaders. Intraclass correlation for the adherence index scores was 0.84 for principals and 0.91 for after-school leaders. Guideline adherence had a wide range in our samples, with an average of 71% for schools and 76% for after-school services. ***Conclusion:* The questionnaires for measuring adherence to the national school meal guideline among primary schools and after-school services are sufficiently reliable for future use in public health evaluation and research**.

## Background

School food environments influence children’s diets [1]. One means to improve these environments is school food guidelines, frequently shown to be effective in improving food availability and children’s dietary intake [2,3]. However, low implementation of school food guidelines and policies is commonly reported [4,5]. Moreover, the lack of valid and reliable assessment tools for evaluating food environments is well documented [6–8], with more work needed, particularly in schools [7]. No validated tools measuring school-level adherence to a comprehensive national school food guideline have been identified in recent reviews [6–8].

In Norway, a revised advisory guideline for food and meals in schools was launched in autumn 2015 [9]. The guideline aims to ensure favourable eating conditions and high nutritional quality of the food and drinks on offer. Norwegian primary schools are obliged to offer after-school care services for schoolchildren in grades 1–4. The food and meal guideline for primary schools applies equally to after-school services. Its 21 recommendations cover organizational aspects of mealtimes (time to eat, supervision, physical and social environments), the nutritional quality of food and drinks on offer, food safety and environmental considerations. Most primary schools offer no food and drinks beyond subscription schemes for fruit and milk, but most after-school services serve one daily meal [10].

School meal practices have been monitored regularly in Norway since the early 1990s through comprehensive mapping surveys issued by the Norwegian Directorate of Health [5]. These were not, however, designed to measure guideline adherence and their psychometric properties were not investigated. Furthermore, the response rates among primary school principals and after-school leaders dropped to 32% in the last surveys in 2013 [5,10], questioning the value of future similar surveys. Shorter and validated questionnaires measuring guideline adherence could potentially increase response rates and would generate valuable data for school nutrition policy making at national or municipal levels. Furthermore, psychometrically sound questionnaires could allow empirical testing of the relationship between school food environments and nutrition outcomes [7].

Guidance on comprehensive approaches to developing questionnaires, including various qualitative and quantitative methods, is available [11]. Cognitive interviewing is a method for improving the content validity of questionnaires by identifying and revising challenging questions through interviews. Interviewers explore whether the information collected reflects the intended content and revise accordingly. Wording, content and design of the questionnaires thereby improve in an iterative manner [12]. Test–retest studies assess the reproducibility of answers in questionnaires. Cohen’s κ and intraclass correlation (ICC) are common reliability parameters for categorical and continuous variables [11], taking variability in the sample into account. The agreement parameter measures only the degree to which scores are identical [13]. By assessing both agreement and reliability, the questionnaires’ potential use in both evaluation and research may be explored [13].

This study aimed to develop two valid and reliable, self-administered, web-based questionnaires to measure adherence to the National Guideline on Food and Meals in School among primary schools and after-school services in Norway.

## Methods

**Figure 1.**
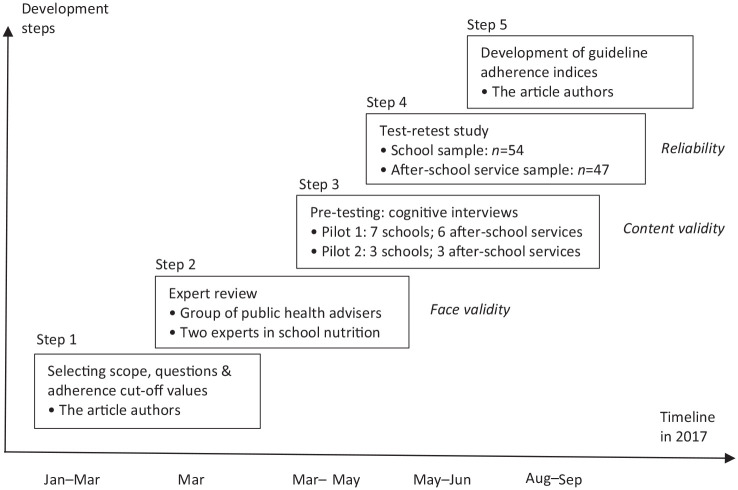
Timeline showing the steps in developing two questionnaires to measure adherence to the national school meal guideline in Norway, including contributors and validation type (in italics).

The process for developing the questionnaires was guided by De Vet et al. [11] and involved both qualitative and quantitative methods (Figure 1). The various study samples are described below. Permission for the study was granted by the Norwegian Centre for Research (NSD) (ref: 52003). All participants received written information about the study, including the right to withdraw at any point. Signed consent forms were obtained from all interview participants. Test–retest participants were informed that answering the questionnaire meant consenting to take part.

### Step 1: Determining scope, questions and adherence values

To limit the response burden, the questionnaire for the principals contained only questions applicable to all Norwegian primary schools, irrespective of food provision. These questions thus covered organizational aspects of mealtimes, access to drinking water, subscription schemes, food safety and hygiene, and availability of unhealthy food and drinks. Similarly, the questionnaire for after-school leaders focused on nutritional quality, food availability, food safety and sustainability. Existing tools were reviewed to guide selection and formulation of questions. Some relevant examples from other countries were identified [14,15], but none aimed at measuring adherence to a food and meal guideline. Some questions from previous Norwegian questionnaires were used in revised versions. For each guideline recommendation, one to seven questions were developed, all with specified cut-off values for adherence. Both questionnaires were in Norwegian.

### Step 2: Expert review of initial drafts

To improve face validity, initial drafts were presented at a 1.5-hour workshop at the Department of Child and Adolescent Health in the Norwegian Directorate of Health and, after revision, were assessed by two experts in food and nutrition in schools.

### Step 3: Qualitative pre-testing with cognitive interviews

To improve content validity, individual cognitive interviews were conducted with principals and after-school leaders in two consecutive rounds of pre-testing. The ‘probing’ technique was utilized, in which interviewers ask follow-up questions during an interview conducted shortly after the participant has completed the questionnaire [12]. Strategic sampling was used to ensure diversity with respect to school size, structure and urban/rural profile, and that schools were recruited from various municipalities in two selected counties, all within a 2-hour drive of Oslo. Schools were invited via a telephone call to principals, who, upon agreeing to participate, received an information letter and were asked to invite the after-school leaders. In the first round of pre-testing (pilot 1), of the 29 schools contacted in Buskerud county nine agreed to participate. Two sites participated with just the principal, and two principals and one after-school leader were excluded because they had not reviewed the questionnaire before the interview. The final sample in pilot 1 consisted of seven principals and six after-school leaders. In round two of pre-testing (pilot 2), the three schools approached in Akershus county all participated with their principals and after-school leaders, yielding 19 complete interviews in total in the pre-testing.

Semi-structured interview guides were developed based on the literature [12,16]. We asked participants to note challenging parts when completing the questionnaires before the interview. During the interviews, we asked them how the questions were interpreted, to elaborate on survey responses and to provide feedback on the challenging parts. Several questions started with: ‘How do you understand. . .?’ and ‘How do you interpret. . .?’ Instead of asking about TV viewing, screen-time or reading out loud when exploring activities during the meal, we asked ‘What activities, if any, take place during the meal?’ The interview guides were revised after pilot 1, to focus on new and adapted questions and response options in pilot 2.

To make commenting easier, paper-based questionnaires were used in pilot 1; in addition, to test functionality, web-based surveys were used in pilot 2. Two researchers participated in all of the 19 complete interviews, one as the moderator with the other taking notes. Participants were informed about the purpose and procedures of the study and the opportunity to withdraw at any time. All participants signed the consent form and agreed to audio recording. We emphasized that our aim with the cognitive interviews was to receive honest feedback on the drafts in order to improve the questionnaires, and not to assess their schools’ adherence to the guideline. After the validity testing, the principal questionnaire had 47 items and the after-school leader questionnaire had 54.

### Step 4: Assessing test–retest reliability

The final pre-tested questionnaires were assessed for test–retest reliability in a nationally representative sample of schools, drawn from an official list of 2392 primary schools. Schools with fewer than 10 children (*n* = 78) and schools that participated in the qualitative pre-testing (*n* = 12) were excluded. Based on the general advice of having about 50 respondents [11], knowledge of typical response rates in test–retest studies and consultation with the Oslo Centre for Biostatistics and Epidemiology, 21% of the 2302 remaining schools were randomly selected from each of Norway’s 19 counties, totalling 483 schools.

Email invitations were sent to principals, who, if agreeing to participate, were asked to forward the invitation to the after-school leaders. The invitations explained the purpose of the study, including why we needed answers to the same questionnaire twice, 8–10 days apart. We explained that participation was voluntary and confidential, and that by answering the questionnaires they consented to participate. Two days after the one-week deadline to respond to the test, a reminder was sent to principals of schools where neither the principal nor the after-school leader had responded, allowing 3–4 more days to respond. To ensure voluntary participation, schools where only one participant had responded by the deadline did not receive reminders. In the retest, both principals and after-school leaders were emailed directly and reminded once if the reply was not received within a few days after the deadline.

### Step 5: Index reliability and adherence levels

To assess the reliability of the questionnaires as a composite score and to determine guideline adherence, a two-step scoring system was developed. First, each respondent obtained a score between 0 and 1 for each relevant recommendation, based on 1–7 questions with equal weighting. Next, these scores were summarized to equal a guideline adherence index score. Schools could reach a maximum score of 12 and after-school services of 15, based on the number of recommendations covered by each questionnaire. By dividing the index score by the number of relevant recommendations, the degree of guideline adherence was determined.

### Analysis

Processing and analysis of the cognitive interviews followed a six-stage model [17]. Relevant sequences of interview data were transcribed from each participant, organized by question, and then compiled for all participants. This procedure was followed in the two pilots for both questionnaires. Both researchers took field notes during the interviews and filled in a structured logbook after each interview, providing contextual information.

Statistical analysis of test–retest reliability was conducted using the program IBM SPSS Statistics 24. Reliability was assessed by calculating Cohen’s κ for nominal variables and quadratic weighted Cohen’s κ for ordinal variables [11]. The κ values can be considered almost perfect at 0.81–0.99, substantial at 0.61–0.80, moderate at 0.41–0.60, fair at 0.21–0.40, slight at 0.00–0.20 and poor if < 0 [18]. Percentage agreement was also calculated for each question. Percentage agreement is considered to be acceptable at ⩾ 70% [13]. Finally, the ICC for absolute agreement (ICC_A_) was calculated to assess the reliability of the adherence index scores. ICC is considered to be acceptable at 0.70 [11].

## Results

The expert review workshop generated two main pieces of advice to improve validity: to reduce the overall scope, and to expect less detailed knowledge from the principals about classroom practices. The two nutrition experts found the revised questions relevant and adequate to cover the guideline’s recommendations but suggested a revised order to improve the flow.

The cognitive interviews lasted around 45 minutes. As shown in Table I, they resulted in many changes to the questionnaires. More revisions were made after pilot 1 than after pilot 2, with the exception being the high number of questions that required both reformulation and new response options in the principal questionnaire after pilot 2. Most of these were minor changes, however, such as changes linked to the splitting up or merging of questions (six cases) or reordering of words in a phrase (three cases).

**Table I.** Types and numbers of changes made during pre-testing to improve content validity of two questionnaires assessing adherence to the school meal guideline in Norway.

Types of changes	Principal questionnaire, no. of changes made		After-school leader questionnaire, no. of changes made	
	After pilot 1	After pilot 2	After pilot 1	After pilot 2
Questions deleted	11	2	10	6
New questions	10	4 (3 were admin)	9	5 (3 were admin)
Merged questions	6 merged with 1 each (thus 6 fewer)	2 merged to 1 (thus 1 less)	3 merged to 1 (thus 2 fewer)	2 merged to 1 (thus 1 less)
Split questions	0	4 split into 3 (thus 8 more)	3 split into 3 (thus 3 more)	1 split into 3 and 1 split into 2 (thus 3 more)
Reformulated questions	0	1	4	5
Questions with new response options	11	5	6	3
Questions reformulated and having new response options	11	14	14	7
Questions with new ordering	3	2	1	3
Change in the total number of questions	7 fewer (from 45 to 38)	9 more (from 38 to 47)	No change: 53	1 more (from 53 to 54)

Some questions were deleted after pilot 1 because they were perceived to be unclear or irrelevant. Two examples of questions with poor clarity were: principals’ interpretation of the teachers’ roles during supervision, and whether the rooms used for eating were ‘physically suitable’. In the after-school services, a question on serving milk with hot meals was perceived as irrelevant because nobody did it. Some unclear phrases were also identified in pilot 2, for example the notion of ‘unwritten rules’ on food brought from home in the principal questionnaire, which was rephrased to ‘oral communication’. Some questions were revised because they presupposed too detailed a knowledge, for example a question to principals on classroom screen use during meals. This was revised twice before a promising solution was identified in pilot 2. In one instance, in after-school services, two rounds of rephrasing could not resolve an interpretation problem, namely that of using ‘lean meat and meat products’.

The two rounds of pre-testing resulted in questionnaires with 47 questions for principals and 54 for after-school leaders. Of these, 27 and 33 questions were used to calculate the adherence indices. The remaining questions were comprised of school background, the respondent’s job position and introductory inquires leading up to the adherence questions, some of which addressed reporting needs among respondents.

In the test–retest study, response rates were 19.3% (*n* = 93) for schools and 18.8% (*n* = 91) for after-school services in the test. Of these, 58 and 52% responded to the retest, respectively, yielding a final sample for the test–retest study of 54 principals and 47 after-school leaders. Both questionnaires had respondents from 18 of Norway’s 19 counties. The average school size was 175 children (range 13–670), which is slightly lower than the national average of 220. The average size of the after-school service was 94 children (range 8–400). Loss to retest was equally distributed geographically across the counties. Only at 14 sites did both the principal and the after-school leader respond. Average administration time in the test and retest study was 12 and 13 minutes for principals and 15 and 11.5 minutes for after-school leaders. Most respondents (80%) in each sample were principals and after-school leaders.

As shown in Table II, κ ranged from −0.03 to 1.0 for the school questionnaire. The reliability rating of the κ values was distributed as follows: 34% perfect or almost perfect, 30% substantial, 24% moderate and 6% fair. No values were rated as slight, two were slightly negative and one could not be calculated. Percentage agreement was ⩾ 70% for 80% of the items, with an average of 85% (range 54–100%). For the after-school questionnaire (Table III), κ values ranged from −0.05 to 0.98 and were rated as follows: 18% perfect or almost perfect, 35% substantial, 25% moderate and 9% fair. Two were rated as slight, two were slightly negative and one could not be calculated. Percentage agreement was ⩾ 70% for 84% of the items, with an average of 82% (range 44–100%).

**Table II.** The kappa (κ) values and percentage agreement for questions included in the school adherence index, organized by corresponding recommendations in the Norwegian National Guideline on Food and Meals in School [9].

Rec^a^	Questions in the principal questionnaire *(response options)*	*n*	κ	Percentage agreement
**1**	Does the school arrange lunch breaks between 10:30 and 12:00 for all pupils? *(yes; no; don’t know)*	53	0.49	96.2
	Does the school offer simple foods (e.g. crispbreads) in cases where pupils do not have a packed lunch? *(No, teacher finds a solution through sharing others’/own food in the classroom. No, students find their own solutions. Yes, pupils are offered food. Don’t know)*	52	0.78	92.3
**2**	Where do the pupils normally eat lunch? *(in the classroom; in the canteen; in the corridor; other place; don’t know)*			
	(a) 1st to 4th grades	53	0.87	98.1
	(b) 5th to 7th grades	49	0.89	98.0
	How often does conversation (no organized activity) constitute most of the lunch break? *(every day; 3 or 4 days/week; 1 or 2 days/week; 1–3 days/month; don’t know)* (a) for 1st to 4th grades	29	0.73^b^	62.1
	(b) for 5th to 7th grades	38	0.83^b^	68.4
	How often does a TV/screen/smartboard constitute most of the lunch break? *(every day; 3 or 4 days/week; 1 or 2 days/week; 1–3 days/month; don’t know)* (a) for 1st to 4th grades (b) for 5th to 7th grades	2926	0.45^b^ 0.66^b^	58.6 53.8
	How does the school define the time used for being present while the pupils eat lunch? *(teaching time; supervision/inspection time)*	49	0.68	85.7
**3**	How much time do the pupils have available for the act of eating? *(< 10 min; 10–14 min; 15–19 min; 20–24 min; 25–29 min; ⩾ 30 min)*			
	(a) in 1st to 4th grades	52	0.74^b^	53.8
	(b) in 5th to 7th grades	52	0.75^b^	59.6
**4**	During how much of the lunch break is an adult present together with the pupils? *(all of it; parts of it; no adult is present during the lunch break)*			
	(a) 1st to 4th grades	53	0.85^b^	98.1
	(b) 5th to 7th grades	52	0.88^b^	96.2
**5**	Do the pupils have access to drinking water in the following ways? *(yes; no, don’t know)*			
	(a) Water from the tap in classrooms/dining rooms	52	−0.03^c^	92.3
	(b) Water dispenser	52	0.93	98.1
	(c) Water fountain	52	0.81	96.2
	(d) Water jugs (in canteen/classroom/dining room)	52	0.56^c^	88.5
	Does the school have a common routine for students’ access to drinking water during class? *(no; yes, common routine that pupils must wait until recess; yes, common routine allowing pupils to drink)*	51	0.33	66.7
**6**	Do the pupils have access to fruit/vegetables/berries at school in any of the following ways?			
	• No access	54	0.89	94.4
	• Yes, free of cost to all pupils	54	0.95	98.1
	• Yes, subscription scheme paid by parents	54	0.95	98.1
	• Yes, fruit/vegetables may be bought in canteen/sales point	54	1.00	100
	• Yes, through a different scheme	54	0.64	94.4
	• Don’t know	54	−^c^	100
	How often are free fruit/vegetables available? *(every day; 3 or 4 days/week; 1 or 2 days/week; 1–3 days/month; don’t know)*	12	1.00^b^	100
	How often is the subscription scheme available? *(every day; 3 or 4 days/week; 1 or 2 days/week; 1–-3 days/month; don’t know)*	11	1.00^b^	100
**7**	How often is milk available? *(every day; 3 or 4 days/week; 1 or 2 days/week; 1–3 days/month; don’t know)*	52	1.00^b^	100
	Are the pupils usually offered the following types of milk? *(yes; no; when there is a need for it; I don’t know)*			
	(a) Whole milk (3.9–4.1% fat, red)	42	0.48	81.0
	(b) Semi-skimmed milk (1.0–1.2% fat, dark pink)	42	0.23^c^	90.5
	(c) Semi-skimmed milk (0.5–0.7% fat, green)	42	0.82	92.9
	(d) Skimmed milk (0.1% fat, light pink)	42	0.23	73.8
	(e) Flavoured milk (raspberry, cocoa)	42	0.81	90.5
	(f) Lactose-free/lactose-reduced milk	42	0.76	90.5
	(g) Juice (apple/orange)	42	0.83	92.9
	(h) Vegetable drinks of soy/oat/almond/rice	42	0.48^c^	85.7
**8**	Roughly how many pupils wash their hands with soap and water before eating? *(nearly all; more than half; about half; less than half; almost none; don’t know)*			
	(a) 1st to 4th grades	49	0.44^b^	87.8
	(b) 5th to 7th grades	48	0.66^b^	70.8
	Does an adult monitor whether pupils wash their hands? *(yes, for all grade levels; yes, but only for 1st to 4th grades; yes, but only for 5th to 7th grades; no)*	50	0.65	80.0
	Does the school have routines for hand hygiene before pupils eat, when on excursions without access to water and soap? *(no, pupils eat without washing hands; no, but bringing disinfectant is encouraged; yes, school/teacher brings disinfectant)*	52	0.52	65.4
**9**	Is the responsibility for controlling that the fridge/cold room remains at the recommended temperature assigned to an adult? *(yes; no; don’t know)*	54	0.59^c^	96.3
	Is the school/after-school service’s food and drink availability, or food handling, reported to the Food Safety Authority? *(yes; no; not relevant; don’t know)*	53	0.75	81.1
**12**	In the course of the year, how often may pupils drink carbonated soft drinks, squash or other beverages containing added sugar or artificial sweeteners during school hours? *(once a week or more often; 1–3 times/month; 5–9 times/year; 3 or 4 times/year; 1 or 2 times/year; never; don’t know)*	53	0.63 ^b^	66.0
**19**	Are birthdays celebrated with cake/ice-cream/sweet buns, etc. during school hours? *(yes, separately for each pupil; yes, a common celebration weekly; yes, a common celebration monthly; no, birthdays are celebrated in ways other than with food; no, birthdays are not celebrated in school; don’t know)*	54	0.43	83.3
	Which of the following foods are used occasionally to reward pupils/classes for good work or behaviour?			
	• We don’t use food as rewards	54	0.63	81.5
	• Chocolate/candy/potato chips, etc.	54	−0.03^c^	92.6
	• Ice-cream/cookies/cake/sweet buns, etc.	54	0.64	83.3
	• Fruit/berries, etc.	54	0.50	81.5
	• Hot dogs/pizza, etc.	54	0.51	85.2
	In the course of the year, how often may pupils eat cake, ice-cream, sweet buns, cookies, etc. during school hours? *(once a week or more often; 1–3 times/month; 5–9 times/year; 3 or 4 times/year; 1 or 2 times/year; never; don’t know)*	54	0.72^b^	61.1
**20**	In the course of the year, how often may pupils eat chocolate, candy, potato chips, etc. during school hours? *(once a week or more often; 1–3 times/month; 5–9 times/year; 3 or 4 times/year; 1 or 2 times/year; never; don’t know)*	53	0.41^b^	77.4

a Rec, recommendations. The full text of the recommendations can be seen in Table IV.

b Weighted κ for ordinal items.

c Values affected by very skewed distribution of answers.

**Table III.** The kappa (κ) values and percentage agreement for questions included in the after-school service’s adherence index, organized by corresponding recommendations in the Norwegian National Guideline on Food and Meals in School [9].

Rec^a^	Questions in the after-school leader questionnaire *(response options)*	*n*	κ	Percentage agreement
**1**	How, and how often, are meals organized after school hours, in the after-school service? *(no meals; 1–3 days/month; 1 day/week; 2 days/week; 3 days/week; 4 days/week; 5 days/week)*			
	(a) Food brought from home	44	0.71^b^	81.8
	(b) Serve bread-based meal	44	0.92^b^	86.4
	(c) Serve hot meal	44	0.82^b^	86.4
**5**	Do the pupils have access to water in the following ways? *(yes; no; don’t know)*			
	(a) Water from the tap in the classroom/dining room	43	0.00^c^	97.7
	(b) Water dispenser	43	0.62	90.7
	(c) Water fountain	43	0.88	97.7
	(d) Water jug	43	0.69	93.0
**6**	How often are fruit/vegetables/berries served in a separate break in the after-school service? *(5 days/week; 4 days/week; 3 days/week; 2 days/week; 1 day/week; 1–3 days/month)*	31	0.73^b^	74.2
	For how many of the bread/crispbread meals are fruit/berries served as a side dish or spread? *(all; almost all; more than half; about half; less than half; almost none; none; don’t know)*	41	0.81^b^	43.9
**7**	For how many of the bread/crisp bread meals in after school service is milk available? *(all; almost all; more than half; about half; less than half; almost none; none; don’t know)*	34	0.85^b^	76.5
	Are the pupils usually offered the following types of milk? *(yes; no; when there is a need for it; don’t know)*			
	(a) Whole milk (3.9–4.1% fat, red)	30	0.29^c^	86.7
	(b) Semi-skimmed milk (1.0–1.2% fat, dark pink)	30	0.61	90.0
	(c) Semi-skimmed milk (0.5–0.7% fat, green)	30	0.79	90.0
	(d) Skimmed milk (0.1% fat, light pink)	30	−0.05^c^	90.0
	(e) Lactose-free/lactose-reduced milk	30	0.34	56.7
	(f) Vegetable drinks of soy/oat/almond/rice	30	0.53	76.7
	How often are the following milk-based beverages available? *(every day; 3 or 4 days/week; 1 or 2 days/week; 2 or 3 days/month; once a month or less often; never; don’t know)*			
	(a) Chocolate milk (e.g. Litago, Sjokomelk)	44	0.54^b^	93.2
	(b) Milk with added cocoa powder (e.g. O’boy, Nesquik)	44	0.72^b^	84.1
	(c) Hot chocolate	44	0.19^b^	79.5
**9**	Where do employees wash their hands before handling/preparing food? *(bathroom sink; kitchen sink used for handwashing and food handling; separate sink for handwashing in the kitchen; don’t know)*	47	0.82	89.4
	Do new employees get a briefing on routines for food safety? *(no; no, but they are asked to familiarize themselves with the rules; yes, they get a walk-through of routines; don’t know)*	47	0.40	68.1
	Is the food handling/food service reported to the Food Safety Authority*? (yes; no; not relevant; don’t know)*	47	0.72	80.9
	Do you have a written protocol for self-monitoring, listing the requirements in the food safety regulation that are relevant to after-school services? *(yes; no; not relevant; don’t know*)	47	0.54	68.1
**10**	Is the food service customized to pupils with food allergy and food intolerance? *(yes, the service ensures that these pupils receive equally good alternatives; no, pupils with food allergy/intolerances must bring their own food; not relevant; don’t know)*	46	0.37^c^	93.5
**12**	Are the following sugar-containing beverages available 1 day per week or more often? *(yes; no; don’t know)*			
	(a) Squash	42	–^c^	100
	(b) Iced tea	42	–^c^	100
	(c) Carbonated soft drinks	42	–^c^	100
	(d) Other beverages containing sugar or artificial sweeteners	42	–^c^	100
**14**	Are the following cereals available 1 day per week or more often? *(yes; no: don’t know)*			
	(a) Breakfast cereals (e.g. 4-korn, muesli)	47	0.83	93.3
	(b) Oats/oatmeal	45	0.82	91.1
	(c) Cornflakes	45	0.62	91.1
	What type of bread is served? *(just whole-grain; more whole grain than white; as much whole-grain as white; less whole-grain than white; just white; don’t know)*	38	0.85^b^	78.9
	What type of crispbread is served? *(just whole-grain; more whole grain than white; as much whole-grain as white; less whole-grain than white; just white; don’t know)*	40	0.55^b^	90.0
**15**	How many different spreads are available during the bread-based meals? *(1 or 2; 3 or 4; 5 or 6; 7 or more; we don’t serve bread)*	47	0.77^b^	83.0
	To what degree is the Keyhole^d^ used when purchasing for selection of healthier spread options? *(to a large degree; to a fairly large degree; to neither a large nor a small degree; to a fairly low degree; to a low degree; don’t know)*	41	0.75^b^	63.4
	During how many of the bread-based meals: *(all; almost all; more than half; about half; less than half; almost none; none; don’t know)* (a) are fish spreads served?	41	0.73^b^	70.7
	(b) are vegetables served as a side dish or spread?	40	0.80^b^	70.0
	(c) is jam served?	41	0.98^b^	85.4
	(d) is chocolate spread served?	41	0.79^b^	97.6
**16**	Is fish served every fifth hot meal or more often? *(yes; no; don’t know)*	36	0.71	86.1
	Is a vegetarian dish (as main) served every fifth hot meal or more often? *(yes; no; don’t know)*	34	0.57	79.4
**17**	Are the following types of butter/margarine usually available to put on bread/crisp bread? *(yes; no; don’t know)*			
	(a) Soft margarine (Brelett, Soft Flora, Vita Hjertego, etc.)	41	0.60	87.8
	(b) Bremykt (semi-soft butter/oil mix)	41	0.64	85.4
	(c) Hard margarine/butter (real dairy butter, Melange, etc.)	41	0.48	95.1
	Are the following types of butter/margarine usually used in cooking? *(yes; no; don’t know)*			
	(a) Oil (rapeseed, sunflower, soy, etc.)	36	0.74	91.7
	(b) Liquid margarine (liquid Bremykt, Melange, etc.)	36	0.48	75.0
	(c) Soft margarine (Brelett, Soft Flora, Vita Hjertego, etc.)	36	0.50	75.0
	(d) Bremykt (semi-soft butter/oil mix)	36	0.63	86.1
	(e) Hard margarine/butter (real dairy butter, etc.)	36	0.54	77.8
**18**	Is salt available for pupils when they eat hot meals? *(yes, they serve themselves; yes, but the amount is supervised by an adult; no; don’t know)*	36	0.53^c^	91.7
**19**	Are birthdays celebrated with cake/ice-cream/sweet buns etc. during the after-school service? *(yes, separately for each pupil; yes, a common celebration weekly; yes, a common celebration monthly; no, birthdays are celebrated in ways other than with food; no, birthdays are not celebrated in the after-school service; don’t know)*	46	0.65	76.1
	In the course of the year, how often may pupils eat cake, ice-cream, sweet buns, cookies, etc. during after-school services? *(once a week or more often; 1–3 times/month; 5–9 times/year; 3 or 4 times/year; 1 or 2 times/year; never; don’t know)*	47	0.52^b^	46.8
**20**	In the course of the year, how often may pupils eat chocolate, candy, potato chips, etc. during after-school services? *(once a week or more often; 1–3 times/month; 5–9 times/year; 3 or 4 times/year; 1 or 2 times/year; never; don’t know)*	45	0.31^b^	60.0
**21**	To what degree is food waste being reduced in the after-school service? *(to a large degree; to a fairly large degree; to neither a large nor a small degree; to a fairly low degree; to a low degree; don’t know; not relevant)*	47	0.53^b^	48.9
	To what degree are environmental concerns taken into account during food service planning? *(to a large degree; to a fairly large degree; to neither a large nor a small degree; to a fairly low degree; to a low degree; don’t know)*	46	0.53^b^	65.2

a Rec, recommendations. The full text of the recommendations can be seen in Table IV.

b Weighted κ for ordinal items.

c Values affected by very skewed distribution of answers.

d The Keyhole is a voluntary Nordic label for food.

Table IV shows the average adherence scores per guideline recommendation, based on the answers from the two samples at test and retest. It illustrates which recommendations are covered by each questionnaire and identifies the most and least adhered to recommendations.

**Table IV.** Average score for each guideline recommendation in test and retest of the school and after-school questionnaires to assess adherence to the Norwegian National Guideline on Food and Meals in School [9].

*The guideline’s 21 recommendations*	*Average score per recommendation*			
	Schools		After-school services	
	Test	Retest	Test	Retest
1. *Meals should be arranged so as to be conducted at 3- to 4-hourly intervals*	0.87	0.89	0.91	0.89
2. *Physical arrangements should be made for meals that promote enjoyment of meals, socialization, well*-*being and health*	0.65	0.65		
3. *Pupils should be given enough time to eat – at least 20 minutes*	0.57	0.54		
4. *Pupils should be supervised by an adult at mealtimes*	0.86	0.87		
5. *Cold drinking water should be available at all times as a thirst quencher and to accompany meals*	0.83	0.76	1.00	1.00
6. *Pupils should be offered schemes that ensure daily access to vegetables, fruit or berries*	0.41	0.41	0.51	0.51
7. *Pupils should be offered schemes that ensure access to milk to accompany meals: reduced-fat semi-skimmed milk (0.7% fat), semi-skimmed milk (1% fat) and/or skimmed milk (0.1% fat)*	0.87	0.80	0.68	0.72
8. *Arrangements should be made to ensure hand washing before meals*	0.65	0.67		
9. *Storage, preparation, serving and labelling of food must be carried out in compliance with rules and recommendations issued by the Norwegian Food Safety Authority*	0.72	0.72	0.51	0.49
10. *Needs of pupils with food allergies or food intolerances should be accommodated*			1.00	1.00
11. *If fruit juice is offered, units should not exceed 250 ml*^a^				
12. *Carbonated soft drinks, squash and other beverages containing added sugar or artificial sweeteners and caffeinated beverages should not be offered*	0.50	0.64	1.00	1.00
13. *Meals offered should be based on the Norwegian Directorate of Health’s healthy eating guidelines and conform to the national nutritional standards*^a^				
14. *Bread and cereals in school meals should be high in fib*re *and whole grains, and low in fat, sugar and salt*			0.91	0.89
15. *Bread toppings/spreads offered to pupils should be varied and always include fish and vegetables*			0.71	0.69
16. *Any hot meals served should be a variety of fish, meat and vegetarian dishes*			0.47	0.45
17. *Cooking oils and liquid and soft margarine should be used instead of hard margarine and butter*			0.57	0.59
18. *Low-salt/sodium foods should be given priority and the use of salt/sodium as seasoning in food preparation and on meals should be limited*			1.00	1.00
19. *Sugary and high-fat baked and other goods should be limited to special occasions*	0.82	0.80	0.80	0.77
20. *Chocolate, confectionery, potato chips and other snacks should not be offered*	0.78	0.72	0.87	0.80
21. *Eco-friendly practices should be aimed for to achieve minimal food waste and meal options in which plant-based foods and fish are focal*			0.35	0.31

a Recommendations 11 and 13 were not included in any of the questionnaires and therefore have no score values.

Among principals, the average obtained adherence index score was 8.3 and 8.2 in the test and retest. For after-school services the scores were 10.5 and 10.4. The ICC_A_ of the adherence index scores was 0.84 (95% confidence interval (CI): 0.73, 0.91) for principals and 0.91 (95% CI: 0.83, 0.95) for after-school leaders. Adjusted to the number of relevant recommendations, adherence levels in the test were 71% (range 42–95%) for schools and 76% (range 53–92%) for after-school services.

## Discussion

This article reports on a comprehensive approach to develop, validate and test the reliability of two questionnaires for measuring adherence to the national school meal guideline in Norway. Cognitive interviews with the target groups increased content validity through improved relevance, wording and user friendliness. The test–retest study demonstrated acceptable reliability for both questionnaires: most items obtained substantial or better κ values, > 80% of items obtained a percentage agreement of ⩾ 70%, and both adherence indices obtained an ICC_A_ > 0.80.

Although some question the extent to which cognitive interviewing may improve validity [12], others suggest that, by identifying faults and improving user friendliness of questionnaires, the method leads to fewer measurement errors and lower response burden [19]. We believe that the types of revisions resulting from our cognitive interviews, coupled with an administration time in the range of 12–15 minutes, provide evidence of increased content validity.

In reliability assessment, reporting both κ values and percentage agreement is recommended [20]. However, De Vet et al. [13] also describe different implications for use of the two parameters; evaluation questionnaires need good agreement and discriminatory questionnaires need good reliability. This is because only κ considers variability, which is important for questionnaires designed to differentiate between units in the sample. In evaluations, measurement error, but not variability, is very important [13]. The κ values demonstrated a large range, reported also in similar studies [14,15,21]. As κ values are heavily affected by skewed prevalence [11], they may be very low despite high percentage agreement. Our results illustrated this: across the two questionnaires only three κ values affected by skewed distributions had an agreement of < 90%. As our questionnaires measure adherence to a guideline that schools should already be implementing, a high number of compliant answers, and thus skewed distribution, are expected. In addition, many questions had few response options, which may reduce the κ values [22]. The few items obtaining both low κ values and agreement should be revised before future use. The higher ICC_A_ for after-school services may reflect the larger number of items in that index, because κ values were slightly better for the school questionnaire. Overall, reliability assessment supports future use of the questionnaires in both research and evaluation.

The main strengths of the present study include the substantial involvement of the target groups in improving content validity, and the relatively large and nationally representative sample of respondents in the test–retest study. A recent review [23] confirms our contention that no prior study has tested the reliability of questionnaires to assess the degree of adherence to a comprehensive national school food guideline. Furthermore, the wide range of adherence levels demonstrates the questionnaires’ potential use in research with discriminative purposes.

The study also has several limitations. First, although cognitive interviewing improved content validity of the questionnaires, additional methods, such as criterion validation through observation, would have assessed validity more robustly. This could have uncovered possible social desirability bias and investigated the extent to which school leaders have sufficient knowledge about classroom practices. Second, although test–retest reliability is essential for new questionnaires [6], an interrater assessment would have been particularly valuable in the absence of a criterion validation. Third, the response rate in the test–retest study was low. However, the repeatability of answers is more important than a representative sample in test–retest studies, as long as the respondents are similar to the intended target group. Finally, the questionnaires for the schools and the after-school services covered only 12 and 15 of the 21 recommendations and therefore did not measure adherence to the entire guideline. Future studies should look at associations between the two adherence indices at each site and between adherence and the school’s socioeconomic profile.

## Conclusion

The results show that the new questionnaires for measuring adherence to the Norwegian National School Meal Guideline are concise, relevant and user friendly, and sufficiently reliable for use in both research and evaluation. Although cognitive interviewing increased the content validity of the questionnaires, firm conclusions about the overall validity could not be drawn.
